# Identification of sacrococcygeal and pelvic abscesses infected with invasive *Mycoplasma hominis* by MALDI‐TOF MS

**DOI:** 10.1002/jcla.24329

**Published:** 2022-03-13

**Authors:** Fang Su, Junwu Zhang, Yongze Zhu, Huoyang Lv, Yumei Ge

**Affiliations:** ^1^ Center of Clinical Laboratory Medicine the Zhejiang Provincial People’s Hospital People’s Hospital of Hangzhou Medical College Zhejiang China; ^2^ Department of Clinical Laboratory Wenzhou Hospital of Traditional Chinese Medicine Affiliated to Zhejiang Chinese Medical University Wenzhou China; ^3^ Key Laboratory of Biomarkers and In Vitro Diagnosis Translation of Zhejiang Province Zhejiang China

**Keywords:** matrix‐assisted laser desorption ionization‐time of flight mass spectrometry (MALDI‐TOF MS), *Mycoplasma hominis* (*M. hominis* MH), puncture fluid, sacrococcygeal and pelvic abscesses

## Abstract

**Background:**

*Mycoplasma hominis* is the smallest prokaryotic microorganism with no cell wall, high pleomorphism, and slower reproduction than bacteria. It is difficult for clinical technicians to find *M*. *hominis* through the negative Gram staining of specimens. Therefore, it is likely to miss detection in routine clinical smear etiological examination. *M*. *hominis* is generally considered to be a common colonizing bacterium in urogenital tract with low pathogenicity, and it is usually difficult to invade submucosal tissue and enter the bloodstream.

**Methods:**

The abscesses of the patient were examined histopathologically, and the pus in the abscesses was extracted for etiological examination. MALDI‐TOF MS was used to identify and confirmed the pathogens in the specimens. The commercial *Mycoplasma* isolation, culture, and drug sensitivity kit was used to determine antibiotic susceptibility.

**Results:**

No pathogens were found after pathological and smear microscopic examination of the puncture fluid from the sacrococcygeal and pelvic abscesses. Until 48 h later, small, translucent, and gray‐white colonies were observed in the blood plate culture results. The laboratory physician ultimately determined that the pathogen was *M*. *hominis* by MALDI‐TOF MS.

**Conclusion:**

We report a case of extra‐urogenital cystic abscesses infected by *M*. *hominis*, in order to improve clinicians’ comprehensive understanding of the pathogenicity of *Mycoplasma*. In addition, the clinical laboratory technician should pay attention to the role of Wright–Giemsa staining of puncture fluid smear in the preliminary detection and the application of MALDI‐TOF MS in identification of uncommon pathogenic microorganisms.

## Graphical abstract

It is generally considered that M. hominis is a low virulence opportunistic pathogen and rarely invades tissue. We report a case of extra‐urogenital cystic abscesses infected by M. hominis, in order to improve clinicians' comprehensive understanding of the pathogenicity of Mycoplasma. No pathogens were found after pathological and smear microscopic examination of the puncture fluid from the sacrococcygeal and pelvic abscesses. Until 48 hours later, small, translucent and gray white colonies were observed in the blood plate culture results. The laboratory physician ultimately determined that the pathogen was M. hominis by MALDI‐TOF MS.

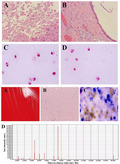

## 1 INTRODUCTION

*Mycoplasma hominis* (*M*. *hominis*) is a common colonization bacterium in the urogenital tract, it can be universally isolated from sexually mature women, approximately 21% to 53% of asymptomatic women are colonized with *M*. *hominis*, and the colonization rate of male urethra can be as high as 20%.^1^, ^2^ Under certain conditions, *M*. *hominis* can cause urogenital tract infections such as pelvic inflammation and cervicitis, but its pathogenicity is weak. Generally, it only causes genital tract mucosal surface infection and does not invade tissues and blood.^3^, ^4^ In this case, the special clinical manifestations of extra‐urogenital cystic abscesses caused by *M*. *hominis* infection were identified by flight mass spectrometry rather than routine Gram staining and colony morphology.

## 2 CASE PRESENTATION

### 2.1 Patient and basic clinical information

**FIGURE 1:**
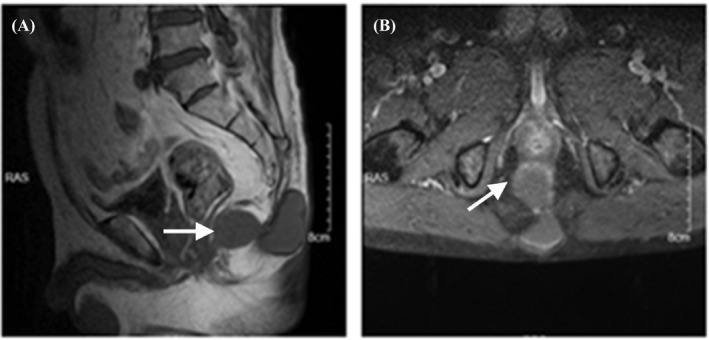
High‐resolution computed tomography (HRCT) of the sacrococcygeal vertebrae showed oval cystic lesions involving in subcutaneous tissue and pelvic cavity. Figure A and Figure B represented the location and size of the abscess under the lateral position and frontal position, respectively. There was an oval cystic lesion near the fifth cone of sacrum and subcutaneous tissue, respectively, and the two lesions were connected. The size of the larger abscess was about 50 × 30 mm, and the size of the smaller abscess was about 40 × 30 mm. White arrow indicated the abscesses

A 70‐year‐old man with more than half a year's history of an oval cystic abscess in sacrococcygeal region presented to the outpatient department with increasingly unbearable pain and fidgety inconvenience. High‐resolution computed tomography (HRCT) of the sacrococcygeal vertebrae showed two connected oval cystic lesions involving in subcutaneous tissue and pelvic cavity (Figure 1). The surgeon performed resection of sacrococcygeal and pelvic abscesses at the request of the patient. The incision was about 7 cm long along the long axis of the cyst, the skin and subcutaneous tissue were cut in turn, the surrounding adhesive tissue was separated along the surface of the cyst, the cyst was completely stripped, the root was ligated with No. 4 silk thread, continued to separate downward, the pelvic abscess of about 40 × 30 mm was stripped, the subcutaneous fascia was free, sutured intermittently, and the incision was closed layer by layer.

### 2.2 Routine smear etiological examination of histopathological and Gram staining

**FIGURE 2:**
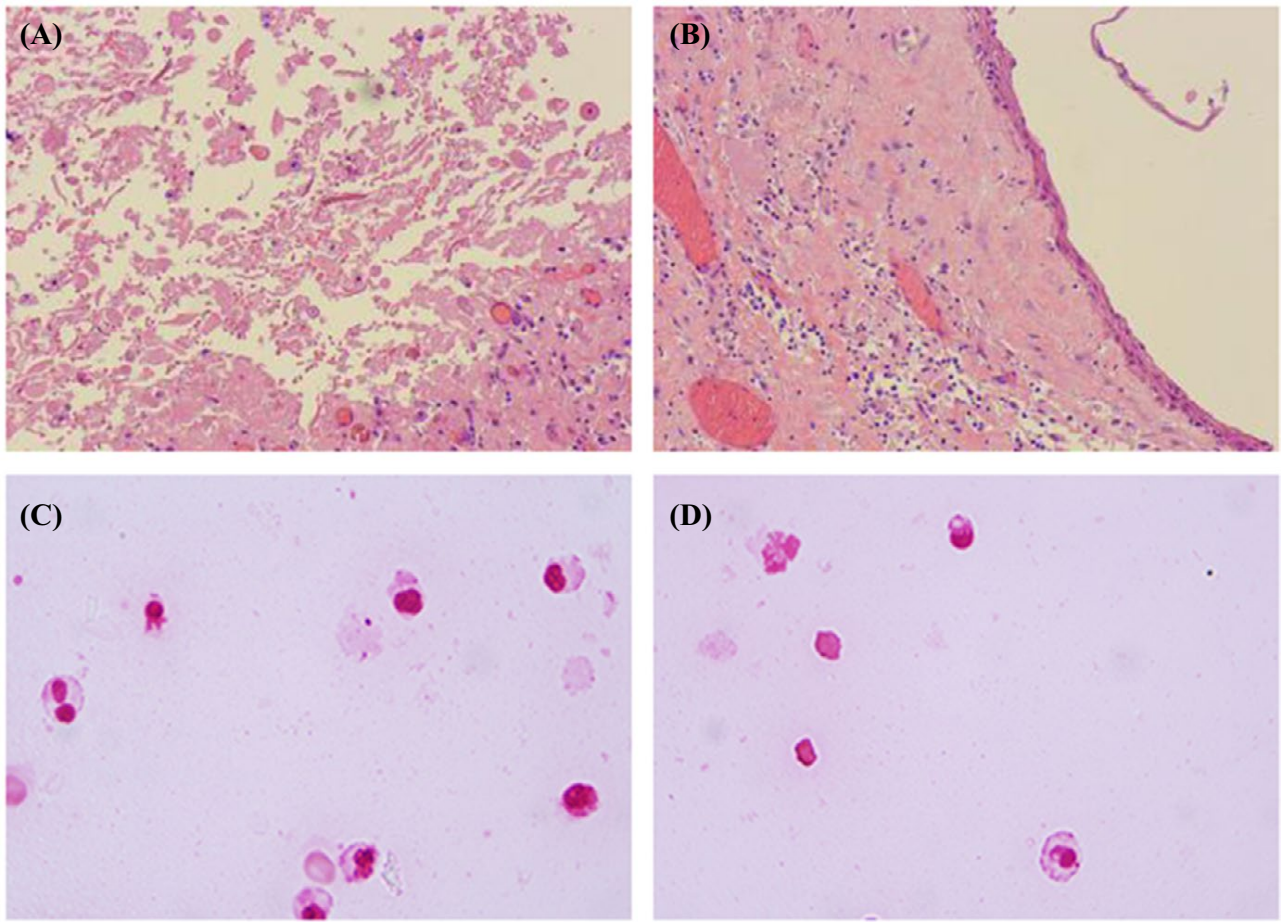
Initial pathogen‐negative results from histopathological examination and Gram staining of abscess tissue and puncture fluid. Figure A and Figure B represented histopathological findings of pelvic and sacrococcygeal abscesses, respectively. The appearance of pelvic abscess was grayish red, the size was 40 × 30 × 14 mm, the section was cystic, the capsule wall thickness was 1 mm, the fibrous capsule wall tissue was accompanied by multifocal lymphocytes infiltration, part of the capsule wall tissue was covered with squamous epithelial cells, the inner wall of the capsule was smooth, and no obvious vegetations were found. The appearance of the sacrococcygeal abscess was grayish red, with the size of 52 × 32 × 25 mm and a cystic appearance in the section, and a brick‐red body in the capsule. The capsule wall thickness was 5 mm, locally calcified. Some cystic wall tissues were covered with squamous epithelial cells, and a small amount of keratinocytes and foam cells could be seen. Figure C and Figure D represented Gram staining results of pelvic and sacrococcygeal abscess, respectively. Both Gram staining results revealed numerous polymorphonuclear leukocytes with no visible pathogens

**FIGURE 3:**
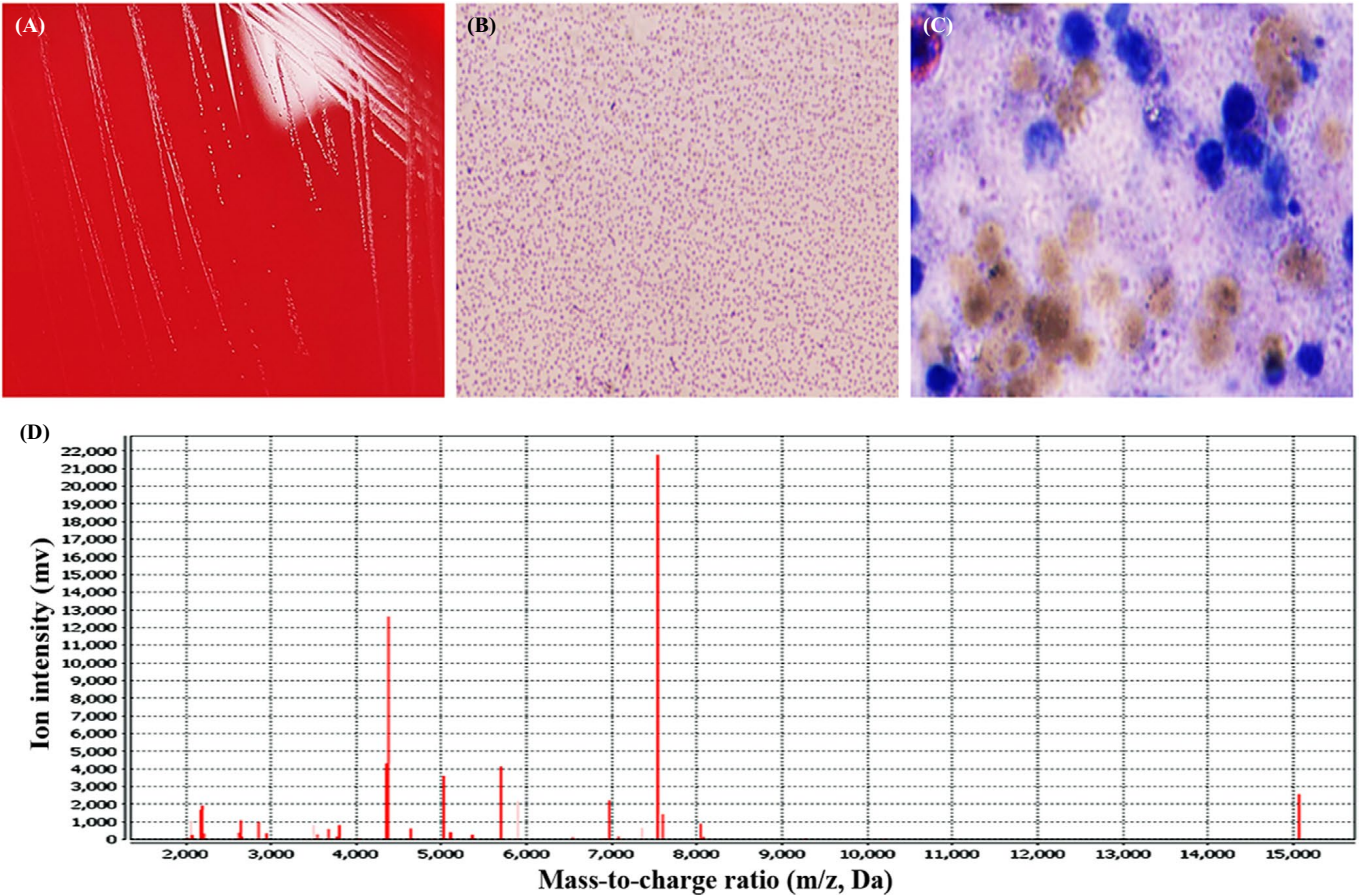
Positive etiological analysis images of colony morphology, Wright–Giemsa staining, and MALDI‐TOF MS detection. Pinpoint‐sized translucent and gray‐white colonies, later identified as *M. hominis*, on blood agar following 48 h of incubation under 5% CO_2_ at 37 °C (Figure A). The colonies on the blood agar plate were stained with Wright–Giemsa staining and small round or rod‐shaped bacteria dyed lavender could be observed under a 100× oil microscope (Figure B). We re‐stained the puncture fluid of the abscess, and Wright–Giemsa staining was used. The results showed that the pleomorphic leukocytes were surrounded by microorganisms of different shapes dyed lavender, with a diameter of about 1–3 mm (100× oil lens and 10× eyepiece) (Figure C). Matrix‐assisted laser desorption ionization–time of flight mass spectrometry (MALDI‐TOF MS) (BioMérieux) was used to identify and confirmed that the pathogen was *M. hominis* with the confidence of 99. 9% (database VITEK MS IVD 3.0 and SARAMIS V4.14) (Figure D)

Both histopathological examination and Gram staining results of the specimens revealed numerous polymorphonuclear leukocytes with no visible pathogens (Figure 2). Pinpoint‐sized translucent and gray‐white colonies were observed on blood agar following 48 h of incubation under 5% CO_2_ at 37 °C (Figure 3A). Microorganisms dyed lavender of different shapes were observed by Wright–Giemsa staining under a 100× oil microscope (Figure 3B). VITEK 2‐compact automatic bacterial detection and analysis system (BioMérieux), one of the most commonly used identification methods of bacteria and Candida in clinic, was failed in this detection.

### 2.3 MALDI‐TOF MS identification and Wright–Giemsa staining results

MALDI‐TOF MS was used to identify and confirmed that the pathogen was *M*. *hominis* with the confidence of 99. 9% (database VITEK MS IVD 3.0 and SARAMIS V4.14) (Figure 3D). Wright–Giemsa staining results of the puncture fluid of the abscesses showed that the existence of the microorganisms of different shapes was confirmed again (Figure 3C).

### 2.4 Antimicrobial susceptibility of the isolated strain

The commercial *Mycoplasma* (*Ureaplasma urealyticum* / *M*. *hominis*) isolation, culture, and drug sensitivity kit (Zhuhai DL) was used to determine antibiotic susceptibility by using the Clinical and Laboratory Standards Institute (CLSI) breakpoints.^5^ The isolate was susceptible to doxycycline, erythromycin, minocycline, and josamycin, while resistant to ciprofloxacin, clindamycin, tetracycline, levofloxacin, ofloxacin, roxithromycin, sparfloxacin, and azithromycin (Table 1). After 3 days of anti‐infection treatment, the patient's condition improved significantly and was discharged after expert evaluation.

**TABLE 1:** Antibiotic sensitivity test results of the isolate from the puncture fluid of the abscesses of the patient

Antibiotics	Quantitative results (μg/ml)	Sensitivity	Methodology	Break point range of antibiotic sensitivity
Ciprofloxacin	≥4	Resistant	MIC	0.25–1
Clindamycin	≥8	Resistant	MIC	Derived from the results
Doxycycline	≤4	Susceptible	MIC	4–6
Erythromycin	≤1	Susceptible	MIC	Derived from the results
Tetracycline	≥16	Resistant	MIC	4–6
Levofloxacin	≥8	Resistant	MIC	Derived from the results
Minocycline	≤4	Susceptible	MIC	4–6
Ofloxacin	≥8	Resistant	MIC	2–8
Roxithromycin	≥8	Resistant	MIC	Derived from the results
Sparfloxacin	≥8	Resistant	MIC	0.5–2
Azithromycin	≥8	Resistant	MIC	2–8
Josamycin	≤2	Susceptible	MIC	Derived from the results

## 3 DISCUSSION

Urogenital inflammation caused by *M*. *hominis* infection mainly includes urinary tract infection, cervicitis, pelvic inflammation, vaginitis, chorioamnionitis, and premature abortion and infertility related to *Mycoplasma* infection. These diseases have been widely reported and are often limited to the surface of urogenital mucosa.^6^, ^7^, ^8^, ^9^ It is generally considered that *M*. *hominis* is a low virulence opportunistic pathogen.^10^, ^11^ Therefore, invasive *M*. *hominis* infection outside the urogenital tract is possible to be ignored by clinicians. What is more worrying is that *M*. *hominis* is gram‐negative due to its lack of cell wall and has no response to all antibiotics targeting cell wall synthesis.^12^ This special biological characteristic makes it can not only escape the routine etiological examination such as Gram staining, but also escape the clinician's empirical anti‐infection treatment. The growth rate of *Mycoplasma* on the blood agar medium routinely used for pathogen proliferation is slower than that of common bacteria, it usually takes 48 h or even longer, and the colony morphology is not typical.^13^, ^14^ Undetected report or untimely pathogen identification may cause an aggravation of the inflammation and lead to poor outcomes of complications and prolonged hospital stays.

At present, the clinical diagnosis of invasive *M*. *hominis* infection outside the urogenital tract is facing great challenges. In this case, clinicians did not tend to consider it as *M*. *hominis* infection at first, and they chose the etiological screening of puncture fluid rather than targeted *Mycoplasma* identification. From the results of histopathological examination and Gram staining, we intuitively found that a large number of leukocytes such as lymphocytes and granulocytes were distributed in the visual field, but there was a lack of visible microorganisms. Therefore, when we encountered such a contradictory phenomenon, the increased number of leukocytes indicated that there was inflammation in this region with a fierce struggle to resist the invasion of bacteria, but there was no colored microorganism, and the infection possibility of pathogenic bacteria with negative conventional staining such as *M*. *hominis* should be considered. In this case, the pathogen of the abscesses was identified as *M*. *hominis* by MALDI‐TOF MS. MALDI‐TOF MS technology is a new soft ionization organic mass spectrometry developed in recent years.^15^, ^16^ By detecting the characteristic protein peaks of the pathogenic microorganisms, the colonies can be identified and classified directly in a few minutes. At present, the flight mass spectrometry database contains 14 main *Mycoplasmas* causing human diseases, such as *M*. *hominis* and M. pneumoniae. This method has the advantages of simple and fast operation, relatively low cost, accurate identification results, and high repeatability, and significantly reduces the cost of consumables and the time of identification and diagnosis. When the traditional colony morphology identification and VITEK 2‐compact automatic bacterial detection and analysis system cannot be determined in clinical laboratory, MALDI‐TOF MS is a favored alternative method.

Extra‐urogenital tract infection caused by invasive *M*. *hominis* should be paid attention to. In recent years, for patients with low immune function or defects, *M*. *hominis* can penetrate the mucosa, invade the vascular walls, and spread to all tissues and organs of the body.^17^, ^18^, ^19^ Surgical incision infection, postoperative blood flow infection, post cardiopulmonary transplantation infection, and other cases have been reported from time to time.^20^, ^21^, ^22^ In addition, infants whose immune function is not yet fully developed often have serious clinical manifestations by infecting *M*. *hominis* in utero or through vertical transmission.^23^ The latest research found that *M*. *hominis* is the main respiratory pathogens in severe novel coronavirus pneumonia in the investigation of SARS‐CoV‐2‐related microbial dysbiosis and various antibiotic‐resistant respiratory pathogens in hospitalized COVID‐19 patients, including 8 mildly and 15 severely ill patients in Guangdong province, China.^24^

## 4 CONCLUSION

Although *M*. *hominis* as a colonization of urogenital tract is common in the human body, attention should be paid to the possibility of invasive *M*. *hominis* infection when patients have chronic diseases such as trauma, diabetes and infectious diseases such as HIV infection, and other immunodeficiency or deficiency conditions, such as tumor and chemotherapy, organ transplant, and immunosuppressive usage. Because the selection of antibiotics for the treatment of *M*. *hominis* infection is highly targeted, timely and accurate etiological results are of great positive significance to prevent antibiotic resistance, shorten the course of disease, and reduce the cost of treatment.

## CONFLICT OF INTEREST

The authors declare that they have no competing interests.

## AUTHORS’ CONTRIBUTIONS

FS collects the patient clinical information. JWZ, YZZ, and HYL analyzed the data. YMG drawn the manuscript. All authors read and approved the final manuscript.

## CONSENT FOR PUBLICATION

Written and informed consent was obtained from the patient for publication of this Case Report and any accompanying images.
